# Sox8 remodels the cranial ectoderm to generate the ear

**DOI:** 10.1073/pnas.2118938119

**Published:** 2022-07-08

**Authors:** Ailin Leticia Buzzi, Jingchen Chen, Alexandre Thiery, Julien Delile, Andrea Streit

**Affiliations:** ^a^Centre for Craniofacial and Regenerative Biology, King’s College London, London SE1 9RT, United Kingdom;; ^b^The Francis Crick Institute, London NW1 1AT, United Kingdom

**Keywords:** ear, gene regulatory network, transcription factor, ectoderm, sensory placode

## Abstract

The vertebrate inner ear arises from a pool of progenitors with the potential to contribute to all the sense organs and cranial ganglia in the head. Here, we explore the molecular mechanisms that control ear specification from these precursors. Using a multiomics approach combined with loss-of-function experiments, we identify a core transcriptional circuit that imparts ear identity, along with a genome-wide characterization of noncoding elements that integrate this information. This analysis places the transcription factor Sox8 at the top of the ear determination network. Introducing Sox8 into the cranial ectoderm not only converts non-ear cells into ear progenitors but also activates the cellular programs for ear morphogenesis and neurogenesis. Thus, Sox8 has the unique ability to remodel transcriptional networks in the cranial ectoderm toward ear identity.

## Significance

Responsible for the sense of hearing and balance, the inner ear is critically important for communication with the environment. In humans, developmental malformations of the ear have lifelong consequences, while age-related hearing defects affect a large proportion of the population. However, many of the underlying genetic mechanisms remain unknown, and no regenerative strategies are available. Here, we characterize the transcriptional and regulatory landscape of ear progenitors, providing unprecedented detail on the molecular aspects of early ear development. We identify the transcription factor Sox8 as a key regulator that initiates the ear program including ear neurogenesis. Our findings not only elucidate how cell fate decisions are regulated in the embryo but can also inform reprogramming and regenerative strategies for the ear.

In the developing embryo, cellular diversity arises through a series of cell fate decisions. Understanding how these decisions take place is therefore a central objective of developmental biology. Cell fate choice is mediated by regulatory factors, which activate a set of transcription factors (TFs) that in turn control the expression of proteins required for cell-specific functions. Direct lineage reprogramming has emerged as a groundbreaking concept, allowing cells to switch fates while bypassing pluripotency, a “shortcut” aimed at improving the speed and efficiency of cell fate conversion (1–3). In turn, lineage reprogramming also highlights the central role of regulatory factors in determining cell fate. Classical examples are the TFs MyoD, which can reprogram fibroblasts into myogenic cells (4), and Pax6, which induces ectopic eyes when mis-expressed in non-eye cells (5–7). Fundamental for perception and interaction with their environment, sense organs and their diversification have enabled vertebrates to thrive in almost every environmental niche. However, apart from Pax6 in the eye, key regulators for other sense organs have not yet been discovered.

Like the eye, the inner ear is a pan-vertebrate sense organ and is responsible for the perception of sound and movement (8–11). During development, it arises from a shared pool of progenitors, which also gives rise to epibranchial neurons. These otic-epibranchial progenitors (OEPs) reside next to the cranial neural plate, where they are intermingled with neural and neural crest precursors (12, 13). Subsequent signaling from adjacent tissues induces the segregation of these fates into distinct territories (14–21). While epibranchial cells produce sensory ganglia, the otic placode invaginates to form a vesicle, which is then transformed into the inner ear, containing many specialized cell types and associated neurons. While signals conferring inner ear identity have been extensively studied, we still lack a comprehensive understanding of the epigenetic mechanisms and TFs regulating its specification.

Here, we model gene expression dynamics during the segregation of otic and epibranchial fates to identify key regulators of ear fate. We use single-cell RNA sequencing (scRNAseq) to determine whether OEPs are progenitors with mixed identity or are prebiased toward their later fate and pseudotime analysis to model transcriptional changes during ear specification. Using epigenomic profiling, we provide a genome-wide identification of ear enhancers and their upstream regulators. Together with functional experiments, we identify a small transcriptional circuit that defines ear identity comprising the TFs Sox8, Pax2, and Lmx1a, with Sox8 at the top of the hierarchy. Sox8 alone triggers the ear program in ectodermal cells and initiates ear morphogenesis by forming ear vesicles containing differentiating neurons. Thus, using a multiomics approach, we have uncovered Sox8 as a critical ear fate determinant and potential reprogramming factor within the developing cranial ectoderm.

## Results

### Dynamic changes in gene expression characterize the transition from progenitor to ear commitment.

**Fig. 1.**
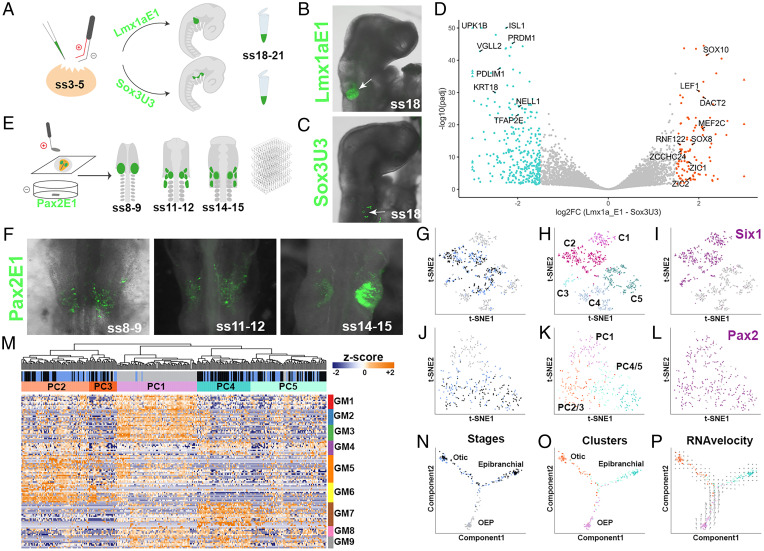
Transcriptomic characterization of ear development. (*A*–*C*) In ovo electroporation (*A*) was used to label and collect otic (*B*; Lmx1aE1-EGFP^+^) and epibranchial (*C*; Sox3U3-EGFP^+^) cells for bulk RNAseq. (*D*) Volcano plot showing genes differentially expressed (absolute log2 fold change > 1.5 and adjusted *P* value < 0.05) in otic (orange) and epibranchial cells (green). (*E*, *F*) Cells expressing the Pax2E1-EGFP reporter active in OEPs and otic and epibranchial placodes were collected for scRNAseq at the stages indicated (*F*). (*G*–*L*) t-distributed stochastic neighbor embedding (tSNE) representation of unsupervised hierarchical clustering of all cells (*G*–*I*) and of the placodal cell subset (*J*–*L*); cells are color-coded according to the stage collected (*G*, *J*; OEP gray, ss11-12 blue, ss14-15 black), clusters (*H*, *K*), and placodal marker expression: *Six1* (*I*) and *Pax2* (*L*). (*M*) Heatmap showing partitioning of the placodal subset (C1, C2 in *H*) into five clusters (PC1–5) based on gene modules (GM). Expression profiles reveal that PC1 largely contains OEP-like cells, while PC2/3 and PC4/5 are composed of otic-like and epibranchial-like cells. (*N*–*O*) Pseudotime ordering using Monocle2 shows trajectories between stages (*N*) and clusters (*O*). Note that otic- and epibranchial-like cells segregate into two branches: otic-like in orange, epibranchial-like in green, and OEP-like in pink. (*P*) RNA velocity vector field verifies the directional trajectories predicted by Monocle2.

To unravel the genetic hierarchy that controls how otic and epibranchial cells diverge, we first characterized the transcriptional profile of cells committed to each fate (22) (Fig. 1 *A*–*D* and *SI Appendix*, Fig. S1 *A*–*F*). To label each cell population, we used two enhancers driving enhanced green fluorescent protein (EGFP): the novel otic Lmx1aE1 enhancer (see Fig. 3 *B*) and the epibranchial enhancer Sox3U3 (23). Reporter constructs were electroporated into the OEP territory of somite stage (ss) 3-5 embryos. At ss18-21, the ear region was dissected and EGFP^+^ cells were isolated by fluorescence-activated cell sorting (FACS) and processed for RNAseq. Differential expression analysis identified 103 and 319 genes up-regulated in Lmx1aE1-EGFP– and Sox3U3-EGFP–expressing cells, respectively (log2FC > 1.5; adjusted *P* value < 0.05; Fig. 1*D* and *SI Appendix*, Fig. S1 *C* and *D* and Dataset S1). This analysis defines the transcriptional states of definitive otic and epibranchial cells.

**Fig. 2.**
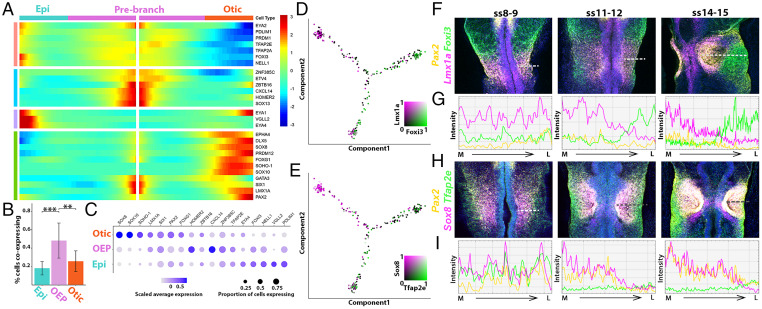
Dynamic gene expression as OEPs segregate into otic and epibranchial fates. (*A*) BEAM identifies genes regulated in a branch-dependent manner. (*B*) Histogram showing the proportion of cells coexpressing genes that are expressed before the branching point but later segregate to the otic or epibranchial branch. Significantly more cells coexpress such genes before the branching point than thereafter (Error bars, ± 1 SD). **P value < 0.01; ***P value < 0.001. (*C*) Dot plot for OEPs and otic and epibranchial markers based on scRNAseq data. Expression level is indicated by color intensity and gene expression frequency by dot size. (*D*, *E*) A proportion of cells coexpresses otic (*Sox8*, *Lmx1a*) and epibranchial (*Foxi3*, *Ap2e*) markers prior to the branching point. (*F*–*I*) In situ HCR (*F*, *H*) and intensity measurements (*G*, *I*) along the medial (M) to lateral (L) axis of the OEP domain indicated by a dashed line in *F*, *H*, confirms gradual segregation of OEP gene expression as distinct otic and epibranchial cell populations emerge.

**Fig. 3.**
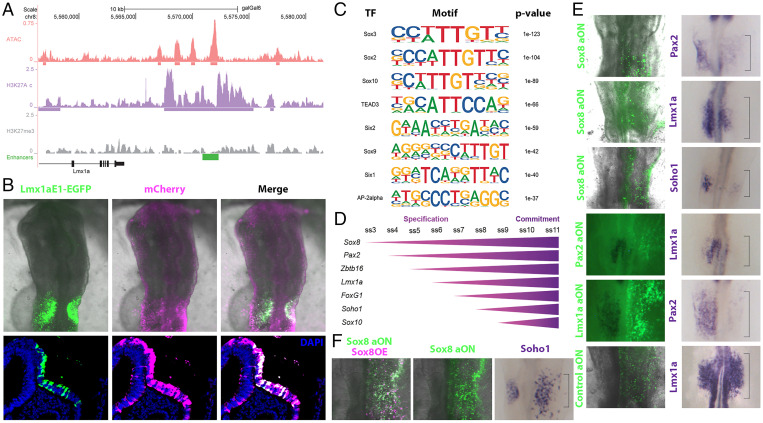
Identification of putative regulatory regions in OEPs. (*A*) Genome browser view of ATAC, H3K27ac, and H3K27me3 profiles in OEPs at the Lmx1a locus. Cloned putative enhancer is shown in green. (*B*) Coelectroporation of Lmx1aE1-EGFP reporter (green) and constitutive mCherry (magenta) reveals in vivo enhancer activity in the otic placode. Sections reveal that the enhancer activity is restricted to the ectoderm at the level of the otic placode: Lmx1aE1 in green, constitutive mCherry in magenta, and DAPI in blue. (*C*) Motif enrichment analysis of all identified enhancers. (*D*) Diagram showing the onset of expression of potential otic regulators during specification and commitment stages. (*E*) Unilateral knockdown of selected TFs using fluorescein-labeled aON (green) leads to down-regulation of otic markers as shown by in situ hybridization (purple) on the targeted side of the embryo. Note: fluorescent images were taken prior to in situ hybridization for Sox8 aON and controls. (*F*) Unilateral coelectroporation of Sox8 aON (green) and Sox8-mCherry construct (magenta) on the right side of the embryo restores *Soho1* expression in the otic territory. Sox8 OE, Sox8 overexpression.

Next, we investigated the transcriptional changes that take place as OEPs transition from a common progenitor population to definitive otic and epibranchial cells. *Pax2* is expressed throughout this time window in both cell populations (*SI Appendix*, Fig. S1 *G* and *H*) (12, 22). Using a Pax2E1–EGFP reporter (see *SI Appendix,* Fig. S5 *A* and *B*), we isolated single cells by FACS from consecutive stages of ear specification — OEP (ss8-9), early-placode (ss11-12), and late-placode stage (ss14-15) – and processed them for scRNAseq (Fig. 1 *E* and *F* and *SI Appendix*, Fig. S1*I* and S2 *B*–*D*). To characterize cellular diversity, we first looked at groups of genes coexpressed across the dataset (gene modules). These gene modules were identified in an unbiased manner through hierarchical clustering of a gene–gene Spearman correlation matrix. Gene modules of interest were selected based on the presence of well-characterized markers for placodal, neural, and neural-crest cells including the new otic and epibranchial genes identified (Fig. 1*D*). Initial cell clustering defined five major clusters (*SI Appendix*, Fig. S2*A*), to which we assigned identities using known markers (Fig. 1 *G* and *H* and *SI Appendix*, Fig. S2 *F*–*I*). Clusters C1 and C2 represented *Pax2^+^/Six1^+^* cells expressing high levels of OEP and placodal makers (Fig. 1 *H* and *I* and *SI Appendix*, Fig. S2 *F* and *I*), while cluster C3 contained contaminating mesoderm (*Twist1^+^*, *Sim1^+^*; *SI Appendix*, Fig. S2*I*). Surprisingly, we also found two clusters with low levels of *EGPF* mRNA (*SI Appendix*, Fig. S2 *A* and *E*) and relatively few *Pax2^+^* cells, one containing neural-like cells (C4; Sox21^+^) and another containing neural crest-like cells (C5; Pax7^+^; *SI Appendix*, Fig. S2 *A*, *G*, *H*, and *I*). Since OEPs are mixed with future neural and neural crest cells at ss8-9 in a *Pax2+* territory (12, 24) and these precursors can coexpress markers for different fates prior to differentiation (25), this observation suggests that while cells in clusters C4/5 initially activate Pax2E1-EGFP, they subsequently down-regulate enhancer activity and *Pax2* expression.

To investigate the transcriptional dynamics accompanying otic and epibranchial fate decisions, we subset the placodal clusters (C1/2 in Fig. 1 and *SI Appendix*, Fig. S2*A*). Reclustering these cells using gene modules containing otic and epibranchial genes, we obtained five placodal clusters (PC1 to 5; Fig. 1 *M*, *J*, and *K* and Dataset S2). PC1 largely consisted of only OEPs (cells collected at ss8-9), while the other clusters contained cells from both early and late placode stages (Fig. 1 *M*, *J*, and *K*). Indeed, PC1 was characterized by the expression of OEP genes (GM1 to 3; Fig. 1*M*) and shared genes with the otic module GM5/6 and the epibranchial module GM7 to 9. In contrast, PC2/3 and PC4/5 were transcriptionally distinct from each other, with profiles akin to otic (GM5/6) and epibranchial (GM7-9) cells, respectively. To explore the relationship between different cell clusters, we organized cells along pseudotime using Monocle2 (26). This analysis predicted that OEPs gradually split into one otic and one epibranchial branch, each composed of early and late placodal cells (Fig. 1 *N* and *O*). Calculating RNA velocity independently (27) and embedding the corresponding vector field onto the Monocle2 trajectory validated the directionality of predicted cell state transitions (Fig. 1*P*).

To explore the dynamic changes of gene expression accompanying these inferred trajectories, we used branch expression analysis modeling (BEAM) (26). This tool identifies groups of TFs expressed in OEPs prior to the branching point that subsequently segregate into either the otic (e.g., *Sox8*, *Lmx1a*, *Pax2*, *Zbtb16*) or the epibranchial (e.g., *Foxi3* (28), *Tfap2a/e*, *Nell1*) branch (Fig. 2*A* and *SI Appendix*, Fig. S3*A*). To quantify the changes in the coexpression of otic and epibranchial genes, we assessed the proportion of coexpressing cells before and after the branching point. A two-tailed Wilcoxon rank sum test revealed significantly more cells coexpressing otic and epibranchial markers in OEPs than in epibranchial (W = 214, *P* = 0.0013) and otic cells (W = 235, *P* < 0.0001) after the branching point (Fig. 2 *B* and *C*). Quantification of gene expression in the monocle trajectories and by in situ hybridization chain reaction (HCR) (29) confirmed that otic (*Sox8*, *Lmx1a*) and epibranchial (*Foxi3*, *Tfap2e*) transcripts overlap at ss8-9 and that their expression resolves as both placodes are firmly established (Fig. 2 *D*–*I* and *SI Appendix*, Fig. S3 *B*–*H*).

Together, these results identify groups of TFs whose expression changes over time as otic and epibranchial precursors segregate and therefore may play a key role in cell fate decisions.

### Epigenomic profiling uncovers regulatory elementsand motifs in ear precursors.

Transcription factors controlling cell fate choice regulate their downstream targets by interacting with tissue specific cis-regulatory enhancer elements. Active enhancers are regions of open chromatin flanked by nucleosomes enriched for histone 3 lysine-27 acetylation (H3K27ac), while actively transcribed genes are marked by histone 3 lysine-4 trimethylation (H3K4me3) (30–33). We therefore profiled ss8-9 OEPs by chromatin immunoprecipitation sequencing (ChIPseq) for H3K27ac, H3K4me3, and the repressive mark H3K27me3 and determined chromatin accessibility by Assay for Transposase-Accessible Chromatin-sequencing (ATACseq). Overlapping H3K27ac and ATACseq data identified 10,969 genomic regions that also showed depleted histone 3 lysine-27 trimethylation (H3K27me3) marks; average profiles showed bimodal H3K27ac read distribution surrounding ATACseq peaks (*SI Appendix*, Fig. S4 *A* and *B*). Of these, just over 70% were intergenic or intronic, representing putative enhancers (8,316), while the remaining were close to transcription start sites (TSSs; *SI Appendix*, Fig. S4*C*). We associated each putative enhancer to the nearest TSS of protein coding genes; gene ontology term analysis of the corresponding genes returned MAP-kinase, Wnt, and Notch signaling known to mediate ear induction, development, and neurogenesis (*SI Appendix*, Fig. S4*D*) (16, 34). To assess their activity in vivo, we selected putative enhancers in the vicinity of ear-enriched genes, generated EGFP reporters, and coelectroporated them with ubiquitously expressed mCherry into head-fold-stage chick embryos. Fluorescence microscopy confirmed enhancer activity in ear progenitors and otic placodes (Fig. 3 *A* and *B* and *SI Appendix*, Figs. S5–S7). To identify upstream regulators that may act as otic determinants, we performed motif enrichment analysis of all 8,316 putative ear enhancers, which revealed an overrepresentation of binding sites for Sox, transcriptional enhanced associate domain (TEAD), and Six family members and for Tfap2a (Fig. 3*C* and *SI Appendix*, Fig. S4*E*). Of these, Tfap2a and Six proteins have previously been implicated in cranial placode development (35–39), confirming that this strategy can identify relevant regulatory factors.

We also exploited the idea that cell identity genes may be regulated by superenhancers characterized by a high density of H3K27ac, while their gene bodies are decorated with H3K4me3 (40–42). Examining OEP TFs that segregate to the ear lineage (Fig. 2*A*), we found that the Sox8 locus was marked with broad H3K4me3 (*SI Appendix*, Fig. S4*F*), while enhancers close to Lmx1a, Zbtb16, and Sox13 were putative superenhancers (Fig. 3*A* and *SI Appendix*, Figs. S4*F*, S6*A*, and S7*A*). These results identify regulatory elements that control gene expression during early ear development as well as several factors that may act as otic specifiers.

### Defining core components of the ear determination network.

Together, the BEAM and epigenomic analysis point to Lmx1a, Zbtb16, and members of the Sox family as potential regulators of ear identity, while previous studies have also implicated Pax2 (43). We next examined the temporal sequence of their expression in otic progenitors. Of the Sox genes, *Sox3* and *Sox13* are expressed prior to ear specification (44, 45), while *Sox9* and *Sox10* are activated later (44, 46). These genes are therefore unlikely to initiate the otic program. In contrast, the SoxE group factor *Sox8* is highly enriched in OEPs prior to segregating to otic cells (Fig. 3*D*). In situ hybridization revealed that *Sox8* begins to be expressed at 3ss, followed shortly thereafter by *Pax2*, *Zbtb16*, and *Lmx1a*, while the known otic factors *Foxg1*, *Soho1*, and *Sox10* are activated later (Fig. 3*D* and *SI Appendix*, Fig. S8*A*).

To explore the regulatory interactions between the earliest OEP TFs, we systematically knocked down each one and assayed the expression of all others as well as that of *Foxg1* and *Soho1* as a readout for otic identity using in situ hybridization (Fig. 3*E* and *SI Appendix*, Fig. S8 *B* and *C*). Control or antisense oligonucleotides targeting Sox8, Pax2, Zbtb16, or Lmx1a were electroporated into future OEPs of head-fold-stage chick embryos, and gene expression was analyzed at OEP stages (ss8-9). We found that Sox8 is necessary for the expression of all assayed ear TFs. Pax2 is required for the expression of *Zbtb16* and *Lmx1a*, which in turn are necessary for *Pax2*, suggesting that they act in a positive feedback loop with Pax2. Zbtb16 is also necessary for *Foxg1*. All gene expression changes can be rescued by coelectroporation of the appropriate full-length constructs (Fig. 3*F* and *SI Appendix*, Fig. S9). Furthermore, transcription factor binding site analysis identified motifs for the core ear network factors (Sox8, Pax2, Lmx1a, Zbtb16) in enhancers associated with *Lmx1a*, *Pax2,* and *Zbtb16* (*SI Appendix*, Fig. S4*G* and Datasets S3 and S4), suggesting that these interactions may be direct. In summary, *Sox8* is the earliest OEP TF and later becomes confined to otic cells. Our functional experiments put Sox8 at the top of the ear determination network forming a regulatory circuit with Pax2, Lmx1a, and Zbtb16.

### Sox8 induces ectopic otic vesicles and vesicle-derived neurons.

**Fig. 4.**
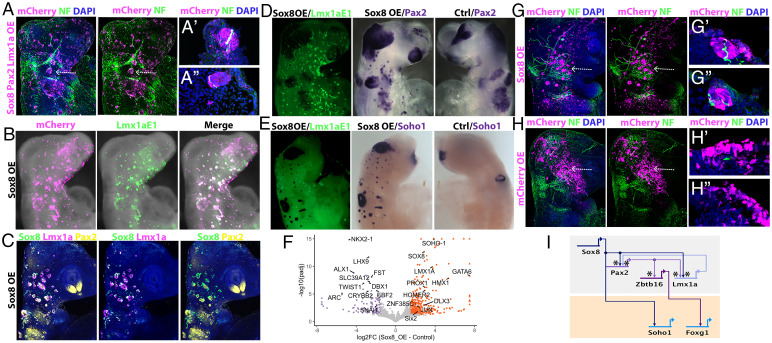
Sox8 converts non-otic cells into otic vesicles with associated neurons. (*A*) Coelectroporation of mCherry-tagged vectors containing the full-length sequence of Sox8/Pax2/Lmx1a (magenta) generates ectopic vesicles across the cranial ectoderm associated with neurofilament-positive neurons (green). (*A’*, *A’’*) Transverse sections at the level indicated by arrows in *A* show neurofilament/mCherry-positive neuronal projections from the ectopic vesicles. (*B*) Overexpression of Sox8 (Sox8 OE; magenta) alone activates Lmx1aE1-EGFP (green). (*C*–*E*) Overexpression of Sox8 (Sox8 OE) induces otic markers on the electroporated side, but not on the control side (Ctrl) as shown by HCR (*C*) and in situ hybridization (*D*, *E*) of otic markers. (*F*) RNAseq of Sox8 and control electroporated cranial ectoderm; volcano plot shows enrichment of otic genes after Sox8OE (absolute log2 fold change > 1.5 and adjusted *P* value < 0.05). (*G*) Sox8-mCherry overexpression (magenta) generates ectopic otic vesicles with neuronal projections (green), while controls do not (*H*); transverse sections of ectopic otic vesicles (*G’*, *G’’*) and control ectoderm (*H’*, *H’’*) show neurofilament-positive neurons within the ectopic vesicle. (*I*) BioTapestry model showing the minimal transcriptional circuit for otic specification with Sox8 at the top of the hierarchy. Asterisks indicate enhancers with predicted Sox8 binding motifs.

If these factors indeed form a minimal circuit driving otic specification, then they should be able to convert non-ear cells into cells with ear identity. We tested this hypothesis by electroporating different combinations of Sox8-, Pax2-, Lmx1a-, and Zbtb16–mCherry–tagged constructs into head-fold-stage ectoderm not destined to contribute to the ear together with the Lmx1aE1–EGFP reporter. We found that misexpression of all four factors and of Sox8/Pax2/Lmx1a activated robust expression of the reporter, while combinations lacking Sox8 did not (*SI Appendix*, Table S7). In addition, Sox8/Pax2/Lmx1a electroporation also resulted in the formation of many *Soho1^+^* otic vesicles scattered across the head ectoderm as well as neurofilament-positive neurons (Fig. 4*A*), the first cell type to differentiate in the otic vesicle (47, 48).

We next asked whether Sox8 alone can initiate the ear program. We found that misexpression of Sox8–mCherry alone activated the Lmx1aE1-EGFP reporter in ectopic vesicles (Fig. 4*B*), as well as the expression of *Pax2*, *Lmx1a*, and *Soho-1* (Fig. 4 *C*–*E* and *SI Appendix*, Fig. S10*A*). To assess the extent to which Sox8 can confer ear identity, we isolated double-positive Sox8–mCherry/Lmx1aE1–EGFP cells from ss11-12 by FACS and compared their transcriptome with a control ectoderm labeled with constitutive mCherry/EGFP. Differential expression analysis showed up-regulation of 399 transcripts in comparison to controls, while 112 genes were down-regulated (log2FC > 1.5; adjusted *P* value < 0.05) (Fig. 4*F* and *SI Appendix*, Fig. S10 *B* and *C* and Dataset S5). Seventeen of the 27 up-regulated TFs are known to be expressed in the inner ear, while for the remaining 10 no expression data were available (Dataset S6). In contrast, among the down-regulated genes were typical neural crest and forebrain transcripts. To confirm that Sox8-expressing cells had indeed acquired otic character, we assessed the expression of otic enriched TFs from previously published data sets (44) in Sox8-overexpressing and control cells (*SI Appendix*, Fig. S10*E*). Of 110 otic TFs, 98 were up-regulated after Sox8 misexpression, but not in controls. This observation suggests that Sox8 alone can confer ear identity to cranial ectoderm cells. Can Sox8 alone also trigger neurogenesis? Indeed, Sox8-induced ectopic vesicles were associated with neurofilament-positive neurites generated from Sox8-expressing cells themselves, while control cells did not form vesicles or neurons (Fig. 4 *G* and *H*).

Together, our results position Sox8 at the top of the otic gene regulatory network (GRN) (Fig. 4*I*), modulating the activity of other ear factors like *Pax2*, *Lmx1a*, and *Soho1*. Downstream of Sox8, Lmx1a and Pax2 seem to form a positive feedback loop with Pax2 required for *Zbtb16* activity, which in turn regulates *FoxG1*. Thus, Sox8 can activate the transcriptional program for ear fate in cells destined to form other sense organs or epidermis.

## Discussion

In this study, we have identified critical components of the ear determination network: Sox8, Pax2, and Lmx1a (Fig. 4*I*). To do so, we have used different criteria, all of which converge on these three TFs: i) temporal sequence of expression, ii) segregation of expression to otic fate using pseudotemporal ordering of single cells and BEAM, iii) position at the top of the otic gene network (44), iv) motif enrichment of newly identified enhancers, v) association with histone marks that define superenhancers and/or fate determinants, and vi) requirement for the expression of known ear markers.

Our analysis puts Sox8 at the top of the transcriptional hierarchy that controls ear fate; Sox8 alone imparts ear identity to cells otherwise destined to form head epidermis, other sense organs ,or cranial ganglia. Previous findings have implicated Spalt4 and other SoxE group family members in otic vesicle formation (49–52). However, their misexpression in chicks or frogs generates only a few ectopic vesicles next to the endogenous ear (49, 50). Unlike Sox8, Spalt4 and Sox10 cannot activate neurogenesis and neuronal differentiation (49, 50), pointing toward a limited ability of these factors to generate functional ear cells from the ectoderm. In mice, loss of Sox8 function does not lead to an overt ear phenotype (53), whereas Sox9 deletion results in defects in placode invagination (51). It is therefore possible that Sox8 and Sox9 play partially redundant roles, although they may also have distinct functions as in other organs (54–57). Thus, the relative importance of SoxE TFs in ear formation may differ across species.

Our experiments propose a prominent role for Sox8 in controlling the ear transcriptional program in chick. Sox8 activates a small regulatory circuit (Fig. 4*I*), which maintains its own expression and as a result otic identity, although ear-specific enhancer analysis (*SI Appendix*, Table S3) suggests that many other downstream effectors may also be direct Sox8 targets. How *Sox8* expression is activated in OEPs remains to be elucidated. While its maintenance requires fibroblast growth factor signaling, its initial expression does not (58, 59). Interestingly, a regulatory element driving *Sox8* expression in the mouse otic vesicle harbors Pax2 binding sites (60), suggesting a positive feedback loop between both factors. In addition, Pax2 is involved in different steps of otic placode formation and patterning (43, 61–63). Sox proteins cooperate with a variety of TFs to exert their cell type specific function (64). It is therefore tempting to speculate that analogous to the eye, where Sox2 cooperates with Pax6 to regulate lens-specific transcription (65), in the ear Sox8 may partner with Pax2.

Finally, our results also show that OEPs initially express competing transcriptional programs that resolve over time as otic and epibranchial cell states are established. We capture previously unknown gene modules that accompany this process as well as regulatory regions associated with otic–epibranchial specification. In turn, this information is critical to unraveling the underlying gene regulatory networks and identifying the TF codes that determine cell identity in the cranial sensory nervous system. In the long term, this will enhance our ability to engineer specific sensory cell types for basic research and regenerative purposes.

## Materials and Methods

### Expression and enhancer constructs.

Putative enhancers were amplified from chick genomic DNA and cloned into pTK-EGFP reporter vectors after digestion with XcmI (66). To generate expression constructs, total RNA was isolated from HH8-12 chick embryos with the RNAqueous-Micro Total RNA Isolation Kit (Thermo Fisher Scientific) and reverse-transcribed using SuperScript II reverse transcriptase (Thermo Fisher Scientific, 180644–014) and oligo-dT primer. Specific primers were used to amplify the full-length coding sequence of Sox8, Pax2, Lmx1a, and Zbtb16, and PCR products were cloned into pCAB-IRES-mCherry. All sequences were verified by Sanger sequencing.

### Chick embryos, electroporation, and culture.

Fertilized hens’ eggs (Stewart, Norfolk, UK) were incubated at 38 °C and staged according to Hamburger and Hamilton (HH) (67). All experiments were performed on embryos younger than 12 d, so they were not regulated by the Animals Scientific Procedures Act 1986.

OEPs for bulk RNAseq were labeled using in ovo electroporation (68); eggs were incubated until the 3–6ss, and pTK-Lmx1aE1-EGFP or pTK-Sox3U3-EGFP plasmids (1 μg/μL) were injected targeting the OEP territory. Electroporation using the Ovodyne electroporator (TSS20, Intracel) was performed with five 50 ms pulses of 8 V at 100 ms intervals. After incubation until ss18-21, embryos were collected in PBS and processed for bulk RNAseq.

For ex ovo culture, embryos were harvested using filter paper rings (69) and cultured on egg albumin; for long-term culture (48 h), the “modified Cornish pasty” method (70) was used. Ex ovo electroporation was performed to collect cells for scRNAseq, for knockdown, rescue, and overexpression experiments. The posterior placodal region or the cranial surface ectoderm of HH6 embryos was targeted for electroporation by injecting plasmid DNA at 1 μg/uL (pTK-Pax2E1-EGFP to label OEPs; pCES-Sox8-mCherry + pTK-Lmx1aE1-EGFP, pCES-Sox8-mCherry + pCES-Lmx1a-mCherry + pCES-Pax2-mCherry; pCES-mCherry + pCES-EGFP for misexpression), control or antisense oligonucleotides (aON; GeneTools; 0.75 mM with 0.3 μg/μL carrier DNA), or a combination of aON and the expression construct (0.75 mM aON + 0.75 μg/μL plasmid). For knockdown and rescue experiments, unilateral injections on the right side of the embryo were performed while the uninjected contralateral left side served as an internal control. Oligonucleotides used were Sox8: 5′-CTCCTCGGTCATGTTGAGCATTTGG-3′ (51); Pax2: 5′-GGTCTGCCTTGCAGTGCATATCCAT-3′ (49); Lmx1a: 5′-CCTCCATCTTCAAGCCGTCCAGCAT-3′ (42); Zbtb16: 5′-GTCAAATCCATAGCACTCCCGAGGT-3′; Sox13: 5′-CTCCTCATGGACATCCATTTCATTC-3′; and control: 5′-ATGGCCTCGGAGCTGGAGAGCCTCA-3′. Electroporation was performed in a chamber using five 5 V pulses of 50 ms in 100 ms intervals (57).

To monitor fluorescence, electroporated embryos were imaged using a Zeiss Axiovert 200M microscope with a Hamamatsu C4742-95 camera and using HC image software. Fluorescent images were taken prior to processing for in situ hybridization and antibody staining.

### Whole-mount and hybridization chain reaction fluorescent in situ hybridization.

In situ hybridization was carried out following previously described protocols (71). Whole-mount pictures were taken using an Olympus SZX12 with a Retiga2000R camera and Q-Capture Pro7 software. Paraffin-embedded embryos were sectioned at 8 μm sections in a Leica RM2245 microtome. Upon sectioning, images were taken in a Zeiss ApoTome.2 coupled with an Axiocam 503 color camera and using the ZEN 2.5 software.

HCR v3 was performed using the Molecular Technologies protocol (29). Briefly, embryos were fixed in 4% PFA for 1 h at room temperature, dehydrated in a series of methanol in PBT, and stored overnight at −20 °C. After rehydration and proteinase-K treatment (20 mg/mL; 3 min), embryos were postfixed in 4% PFA for 20 min. Embryos were then washed on ice in PBS, 1:1 PBT/5× SSC (5× sodium chloride sodium citrate, 0.1% Tween-20), and 5× SSC for 5 min each. Prehybridization in hybridization buffer was performed for 5 min on ice, followed by 30 min at 37 °C. Embryos were hybridized overnight at 37 °C with probes at 4 pmol/mL in hybridization buffer. After four 15 min washes with a probe wash buffer at 37 °C, preamplification was carried out in amplification buffer for 5 min at room temperature. Hairpins were prepared individually at 30 pmol final concentration; they were incubated at 95 °C for 90 s followed by cooling to room temperature for 30 min, protected from light. Cooled hairpins were added to 500 μL amplification buffer, and embryos were incubated in hairpins overnight at room temperature followed by two 5 min and two 30 min washes in 5× SSC. After a 5 min incubation in DAPI (10 mg/mL), they were washed 3 times for 10 min with 5× SSC before being imaged using a Leica SP5 laser scanning confocal inverted microscope using the LAS AF software.

For HCR image analysis in Figs. 2 and 4, Z-stacks were collected for 50 to 70 μm; figures show projections of all stacks. Images were processed using ImageJ, and the ImageJ Plot Profile tool was used to calculate intensity plots. In brief, an optical section in the center of the placode territory was selected using the Pax2 channel as a reference. The Pax2 channel was added to the Sox8, Lmx1a, Foxi3, or Tfap2e channels, respectively. Intensity values were then calculated across the area of interest and plotted.

### Whole-mount immunostaining.

Embryos were collected in PBS, fixed for 25 min at room temperature in 4% PFA, washed in PBS-Tx (PBS + 0.2% Triton X-100), and blocked in 5% goat serum in PBS-Tx for 3 to 5 h at room temperature. Embryos were then incubated in primary antibody diluted in blocking buffer for 24 to 72 h at 4 °C. Primary antibodies were rabbit anti-mCherry (1:200; Abcam ab167453), mouse anti-NF (1:100; Thermo Fisher Scientific 13–0700), rabbit anti-GFP (1:500; Abcam a11122), or mouse anti-GFP (1:1,000; Molecular Probes A11120). After five 60 min washes and one overnight wash in PBS-Tx, embryos were incubated in secondary antibodies (1:800) at 4 °C overnight. The secondary antibodies used were goat anti-rabbit IgG Alexa Fluor 488 (Thermo Fisher Scientific, A11036), donkey anti-rabbit Alexa Fluor 568 (Thermo Fisher Scientific, A11001), goat anti-mouse IgG Alexa Fluor 488 (Molecular Probes A11001), and goat anti-mouse IgG Alexa Fluor 568 (Molecular Probes A11004). Embryos were then briefly incubated in PBS containing 10 mg/mL DAPI and washed at least 5 times in PBS-Tx before being mounted on slides and imaged using the Leica SP5 laser scanning confocal inverted microscope with a 10× objective (Figs. 2 and 4 *A, C, G, and H*) or an Olympus SZX12 with a Retiga2000R camera and Q-Capture Pro7 software (Figs. 1, 3, and 4). Confocal whole-mount images are maximum intensity projections of embryo z-stacks. Sections were imaged using a 63× oil immersion objective, and maximum intensity projections are shown.

### Cryosectioning.

Embryos were embedded in gelatin as previously described (72) and cryosectioned at 15 to 20 μm using a Bright OTF5000 cryostat. Sections were mounted using Mowiol 4–88 (Sigma Aldrich, 81381) and imaged using the Leica SP5 laser scanning confocal inverted microscope (LAS AF software) or a Zeiss Axiovert 200M microscope with a Hamamatsu C4742-95 camera and using OCULAR software.

### Bulk RNA sequencing.

To label otic and epibranchial cells, embryos were electroporated with Lmx1aE1-EGFP and Sox3U3-EGFP plasmids, respectively, and whole heads were used for cell collection. For overexpression experiments, embryos were electroporated with Sox8-mCherry + Lmx1aE1-EGFP or with pCAB-mCherry + pCAB-EGFP, and the endogenous otic placode and the trunk were removed before cell dissociation. Cells were dissociated in FACSmax cell dissociation solution (Ambion, T200100) containing papain (30 U/mg, Sigma-Aldrich, 10108014001) for 20 min at 37 °C before being transferred to HBSS without Ca and Mg (HBSS, Life Technologies, 14185045) containing 5% heat-inactivated FBS, rock inhibitor (10 μM, Stemcell Technologies, Y-27632), and nonessential amino acids (Thermo Fisher Scientific, 11140035). Cells were disaggregated by pipetting, sequentially filtered through 0.35 μm and 0.20 μm filters (Miltenyi Biotech, 130–101-812). Pelleted cells were resuspended in 500 μL HBSS and isolated by FACS using a BD FACS-Aria Diva. Next, 2,000 cells per biological replicate were collected, centrifuged at 200g for 5 min at 4 °C, washed with PBS, and resuspended in lysis buffer. RNA was extracted using the Ambion RNAqueous Micro Total RNA isolation kit (AM1931, Thermo Fisher Scientific). RNA integrity was checked using the Bioanalyser with Agilent RNA 6000 pico kit (Agilent Technologies, 5067–1513); samples with a RNA integrity number (RIN) > 7 were processed for library preparation. Sequencing libraries were prepared using the Nextera Sample low input kit (Illumina, 15028212) and sequenced using 75 bp paired-end reads on the Illumina Hiseq4000 platform. A minimum of 3 biological replicates were used for analysis.

### Single-cell RNA extraction, library preparation, and sequencing.

HH6-7 embryos were electroporated with Pax2E1-EGFP to label OEPs; cells were dissociated as described above ,and 288 EGFP+ cells from each ss8-9, ss11-12, and ss14-15 were collected by FACS in 96-well plates. Sequencing libraries were prepared following the SmartSeq2 protocol (73). Libraries were sequenced on the Illumina NextSeq500 platform using single-end 75 bp sequencing (ss8-9) or on the HiSeq4000 platform using paired-end 75 bp sequencing (ss11-12, ss14-15).

### FACS of cells.

Cells were collected using a BD FACS-Aria Fusion. For scRNAseq, three batches of experiments were performed, one per stage. Live EGFP+ cells were selected using propidium iodide (for ss8-9 cells) or DAPI (ss11-12, ss14-15) as a live/death cell marker. The gating tree was set as follows: first FSC-A/SSC-A, which represented the distribution of cells based on size and intracellular composition, respectively. Then either FSC-A/FSC-H (ss8-9) or SSC-W/SSC-A (ss11-12, ss14-15) was used to exclude the events that might represent more than one cell. Next, we performed a live gate to select the cells that were propidium iodide/DAPI-negative. Finally, GFP+ cells were identified and selected for sorting. For bulk experiments, we used DAPI as a live/death marker and gating was performed as described above for ss11-12/ss14-15. For Sox8OE/Lmx1a-E1+ and mCherry/EGFP+ control cells, the last step of the gating tree was performed using GFP/mCherry to select the double-positive population. In all the experiments, a 100 μm nozzle and 20 psi pressure were used.

### Nuclei isolation, ATAC library preparation, and sequencing.

The ATACseq library was prepared following published protocols (74). Approximately 30 pieces of the OEP territory were dissected from ss8-9 embryos and dissociated with Dounce homogenizer (tight pestle) in lysis buffer (10 mM Tris⋅HCl, pH7.4, 10 mM NaCl, 3 mM MgCl2, 0.1% IGEPAL CA-630). Nuclei were pelleted at 4,000 rpm at 4 °C for 10 min. After removing the lysis buffer, 1.25 μL Tn5 transposase (Illumina, FC-131-1024) was added to 25 μL reaction volume and incubated at 37 °C for 10 min. Tagmented DNA was then purified with the Mini Elute PCR purification kit (Qiagen, 28004) followed by 9 cycles of PCR enrichment using the NEB High-fidelity PCR kit (NEB, M0541S). The quality of ATAC libraries was assessed using the Agilent Bioanalyzer with the DNA High Sensitivity kit (Agilent Technologies, 5067–4627) and quantified with the Kapa NGS library quantification kit (Kapa Biosystems, KK4824). The libraries were sequenced with the Illumina HiSEq. 2000/2500 in 2 × 100 cycles.

### H3K27ac, H3K27me3, and H3K4me4 ChIP library preparation and sequencing.

Approximately 200 pieces of OEP ectoderm were dissected from ss8-9 embryos. The tissue was dissociated in nuclei expulsion buffer (0.5% Nonidet P-40, 0.25% Triton X-100, 10 mM Tris⋅HCl, pH 7.5, 3 mM CaCl2, 0.25M sucrose, 1× protease inhibitor [Roche], 1 mM DTT, and 0.2 mM PMSF) using a Dounce homogenizer (loose pestle); cross-linking was performed in 1% formaldehyde for 9 min, followed by quenching with 1M glycine for 5 min. Cells were then pelleted and washed 3 times with PBS containing a protease inhibitor (Roche, 11873580001). The pellets were snap-frozen and stored at −80 °C for later use. Cross-linked chromatin was fragmented by sonication in an ice bath (Misonix Q700 at 7 amplitude, 5 × 40 s, 30 s on, 60 s off) and immunoprecipitated following the Nano-ChIP protocol (75). For ChIP, the following antibodies were used: anti-H3K27Ac (Abcam, ab4729), anti-H3K4me3 (Diagenode, A5051-001P), and anti-H3K27me3 (Millipore, 07449). Adaptors and primers from the NEBNext library preparation kit (Illumina, E6040S) were used to prepare the library following the NanoChip protocol (75). The library was enriched using 14 PCR cycles and quantified with the Kapa NGS library quantification kit (Kapa Biosystems, KK4824) before pooling at a concentration of 20 nM. Library quality was assessed using the Agilent Bioanalyzer with the DNA High Sensitivity kit (Agilent Technologies, 5067–4627) and quantified with the Kapa NGS library quantification kit (Kapa Biosystems, KK4824). The libraries were sequenced with Illumina HiSEq. 2000/2500 in 2 × 100 cycles.

### High-throughput sequencing-data analysis.

All data alignment and downstream analysis was carried out using NF-core and custom Nextflow pipelines to allow full reproducibility. All code used, including Nextflow pipelines and downstream analysis, can be found at https://github.com/alexthiery/otic-reprogramming. Full detailed documentation for the pipeline is also available at https://alexthiery.github.io/otic-reprogramming/. A custom Docker container used for the downstream analysis pipeline can be found at https://hub.docker.com/repository/docker/alexthiery/otic-reprogramming-r_analysis. This also allows for interactive exploration of the data.

### Bulk RNAseq.

Bulk RNAseq data were processed and aligned to GalGal6 using the default NF-core RNAseq (v2.0) pipeline (76), which uses the STAR aligner. Downstream differential expression analysis (Lmx1aE1-EGFP vs. Sox3U3-EGFP; Sox8OE vs. ControlOE) was carried out with the DESeq2 package in R (77). Adjusted *P* values were calculated using the default DESeq2 multiple test correction (Benjamini–Hochberg). Differentially expressed transcripts were determined by an absolute log2 fold-change >1.5 and adjusted *P* value < 0.05.

### scRNAseq alignment.

SmartSeq2 scRNAseq reads were aligned and processed using a custom Nextflow DSL2 pipeline. Adaptor sequences were trimmed using Cutadapt (v2.10). HISAT2 (v2.2.1) was then used to build a genome index from GalGal6 (amended to include a GFP sequence) before extracting splice sites from the GTF and aligning reads. Read counts were obtained using HTSeq (v0.12.4). BAM files from HISAT2 were also passed to Velocyto (v0.17) (27) in order to get spliced and unspliced expression matrices for further downstream analysis.

### scRNAseq data analysis.

Downstream data analysis was carried out primarily using the Antler R package (version: Development2019) (78). We excluded from the dataset cells that expressed fewer than 1 k genes or fewer than 500 k reads, cells with more than 6% reads from mitochondrial genes, genes that were expressed in fewer than 3 cells, and genes with counts per million reads mapped CPM < 10.

### Identification of gene modules.

To identify clusters of genes with correlated expression in an unbiased manner (gene modules), we used the Antler identifyGeneModules function. Genes that did not have a Spearman correlation > 0.3 with at least three other genes were first removed. Genes were then iteratively hierarchically clustered into gene modules and filtered. Gene modules were filtered based on the minimum expression level (5 CPM) and the proportion (0.4) of cells expressing a gene module. The number of final gene modules was set to 40 to achieve reasonably large gene modules, which broadly characterized cell type diversity across the dataset. These gene modules were then filtered based on the presence of genes with known expression profiles in ectodermal derivatives. Cells were then reclustered based on the remaining gene modules, with the expression of known markers used to assign cell states. Placodal cells were then subset and reclustered using a new set of gene modules identified from the subset cell population. Three main cell states were identified as otic, epibranchial ,and OEP populations.

### Gene expression dynamics at the otic-epibranchial branching point.

To model the transcriptional dynamics at the otic-epibranchial branching point, we ordered cells along pseudotime using Monocle2 (79). The lineage tree was rooted to the earliest cell state (ss8-9), which comprised mostly OEPs. The expression of genes along the bifurcation point was then modeled using BEAM.

To assess whether individual OEPs simultaneously express markers associated with the otic or epibranchial state, we calculated the coexpression of key otic and epibranchial genes within each of the Monocle branches. First, gene expression was binarized based on the presence or absence of gene expression; then the proportion of cells coexpressing pairs of otic-epibranchial genes within each of the branches was calculated. A Kruskal–Wallis test was then used to compare the proportion of cells coexpressing otic and epibranchial genes between the three branches. Posthoc pairwise comparisons between the OEP branch and the otic or epibranchial branch were carried out using 2-tailed Wilcoxon rank sum tests.

RNA velocity provides an alternative to Monocle2 for inferring the future cell state of individual cells. Spliced, unspliced, and spanning matrices obtained from Velocyto (27) were subset in R based on genes and cells used to generate the Monocle trajectory. RNA velocity was then calculated using the Velocyto.R package, where spanning reads were used to fit gene offsets. Single-cell velocities as well as vector fields were subsequently visualized on both tSNE and Monocle DDRTree embeddings.

### ChIPseq and ATACseq alignment and peak calling.

ChIPseq and ATACseq data were processed and aligned to GalGal6 using the default NF-core ChIPseq (v1.2.0) and NF-core ATACseq (v1.2.0) pipelines (76), respectively. Reads were aligned using the Burrows–Wheeler aligner. MACS v2.2.7.1 was used to call broad peaks (false discovery rate [FDR] < 0.1) and narrow peaks (FDR < 0.05) for ChIPseq and ATACseq data, respectively.

### Enhancer discovery.

Putative enhancers were identified using a custom Nextflow DSL2 pipeline. First, bedtools (v2.29.2) was used to subset ATACseq peaks that overlapped with an H3K27ac peak while removing those overlapping with H3K27me3 peaks. The remaining ATACseq peaks were then annotated with Homer (v4.11), using a GalGal6 GTF that was filtered for protein coding genes. Promoter and exonic peaks were then removed. Motif enrichment and functional enrichment analysis were carried out using Homer and g:Profiler, respectively.

### Transcription factor binding site prediction.

Enhancers that showed EGFP reporter activity in the otic placode were scanned for TF binding sites using the RSAT matrix scan tool, which scans DNA sequences with position-specific scoring matrices (rsat.sb-roscoff.fr/matrix-scan_form.cgi). A curated list of matrices was used (Dataset S4). Both strands were scanned specifying the background model estimation for organism-specfic (Gallus gallus) and asking to return motifs with a *P* value < 0.001.

## Supplementary Material

Supplementary File

Supplementary File

Supplementary File

Supplementary File

Supplementary File

Supplementary File

Supplementary File

## Data Availability

All material will be made available upon request after appropriate material transfer agreements. All data have been deposited in Gene Expression Omnibus (accession number GSE168089) (80). Nextflow pipelines and downstream analysis can be found at https://github.com/alexthiery/otic-reprogramming (81). Full detailed documentation is available at https://alexthiery.github.io/otic-reprogramming/ (82). A custom Docker container used can be found at https://hub.docker.com/repository/docker/alexthiery/otic-reprogramming-r_analysis (83).
